# Ultrasound-aided pH-shift processing for resource-smart valorization of salmon and herring side streams

**DOI:** 10.1016/j.ultsonch.2023.106539

**Published:** 2023-08-01

**Authors:** Michaela V. Santschi, Ingrid Undeland, Mehdi Abdollahi

**Affiliations:** a Department of Health Science and Technology, Institute of Food, Nutrition and Health, ETH Zurich, 8092 Zurich, Switzerland; b Department of Life Sciences – Food and Nutrition Science, Chalmers University of Technology, SE 412 96 Gothenburg, Sweden

**Keywords:** pH-shift method, Alkaline solubilization, Isoelectric precipitation, Fish co-products, Protein ingredient, Ultrasound, Water footprint

## Abstract

The possibility of reducing the amount of fresh water used during alkaline the pH-shift processing of salmon head (SH) and herring frame (HF) was evaluated with ultrasound (US) as a tool to mitigate its negative effects on protein yield. The role of water ratio and US for homogenate viscosity, mass yield, crude composition, functional properties and lipid oxidation of the SH and HF protein isolates were also investigated. Applying US during the solubilization step of the pH-shift process completely compensated for the reduced protein yield coming from using 3 rather than 6 volumes of water for HF, but not for SH. Using US had no negative effect on the composition and protein functionality of the HF protein isolate. However, it slightly increased its level of secondary lipid oxidation products. Altogether, applying US during the pH-shift processing at low water ratios can be a promising solution for more resource-smart valorization of herring side streams.

## Highlights

• Ultrasonication during protein extraction enabled reducing water ratio to half.
• The effect of ultrasound on the yield was dependent on the type of side stream.
• Applying ultrasound did not alter protein functionality within the same water ratio.
• Ultrasonication slightly increased lipid oxidation in the recovered proteins.

## 1 Introduction

While the world population and its awareness about the benefits of seafood have been increasing, the demand for fish and seafood products has simultaneously raised [1], [2]. However, these demands can no longer be met with traditional resources and have led to overfishing [3]. This has caused great interest in using underutilized resources such as side streams from industrial seafood processing for food production [2]. Currently, the global seafood supply chain suffers from tremendous inefficiency during processing, in fact up to 70% of all aquatic resources entering steps like filleting and peeling end up as side streams [1]. Mostly these side streams, including head, frame and viscera, end up in low-value feed production or even as waste [4], despite the fact, that they are rich sources of high value food grade nutrients such as fish protein, oils, vitamins and minerals [5]. However, due to their high content of pro-oxidative heme-proteins and sensitive lipids as well as their bony nature, successful use of these valuable raw materials is often very challenging [5].

A promising method to recover proteins from complex biomasses, while maintaining their functionality, is the so-called pH-shift process. In the process, which is run below ambient temperature, proteins are selectively extracted from the raw material by homogenizing it in 6 to 10 ratios of water at high (>10.5) or low (<3.5) pH followed by centrifugal separation. Thereafter, the solubilized proteins are recovered by isoelectric precipitation (pH ∼ 5.5) and finally dewatered by centrifugation or filtration [6], [7]. Compared to other valorization technologies e.g., enzymatic hydrolysis, the pH-shift technology is energy-smart as there is no need for drying of the final product. Further, it consists of intact polypeptide chains that are able to form strong gels. However, at present the pH-shift process still requires large amounts of fresh water which in an industrial scale can result in 6′000-10′000 L of wastewater per ton of processed fish side stream. The extensive use of water also yields large volumes of feed which must pass through the process line, increasing process time. Consequently, the process gets very costly from both economic and environmental perspectives. Thus, strategies for engineering the pH-shift process to make it more resource-smart in terms of water consumption must be investigated.

Simply decreasing the ratio of water, i.e., the main solvent in the process, will bear the risk of decreasing the protein yield which jeopardizes the economic feasibility of the process. This stems from reduced protein solubilization and from large partitioning of proteins into the sediment of the first centrifugation step. For instance, Chomnawang and Yongsawatdigul [8] reported a decreased protein extractability from tilapia frame when reducing the raw material- to-water ratio from 1:9 to 1:7, 1:5 and 1:3 in the alkaline version of the pH-shift process. We hypothesize that introducing an additional assistant technology, namely ultrasound (US), to the pH-shift process can compensate for the negative effects on yield caused by decreasing the water ratio. High-intensity US is perceived as an ecological and economically viable food processing technology as it has shown to reduce the energy consumption and shorten the processing time of extraction processes [9]. US-induced process effects are mainly attributed to the cavitation phenomenon, which promotes permeation of solvents into the raw material and increases its interaction with proteins. This consequently increases mass transfer and internal diffusion mechanisms [10], [11]. It has been shown in a few studies that adding an US step during pH-shift processing of marine resources provides increased protein solubility and total protein recovery [10], [12], [13]. However, to the best of our knowledge, no research exists investigating the possibility of combining the US technology with the pH-shift process in order to reduce the amount of water needed in the process.

The present study was aimed to investigate if US-assisted protein solubilization can mitigate the expected yield-lowering effects of reducing the water ratio during pH-shift processing of salmon head (SH) and herring frame (HF) without jeopardizing crude composition, lipid oxidation and functional properties of protein isolates. In the study, the raw material-to-water ratio was reduced from a standard of 1:6 to 1:4 and to 1:3 while an additional US step was introduced to the process.

## 2 Material and methods

### 2.1 Chemicals

All chemicals used were reagent grade and purchased from Sigma-Aldrich (Steinheim, Germany) or Merck KGaA (Darmstadt, Germany) if not indicated otherwise. Hydrochloric acid (pure, 37% solution in water), ammonium thiocyanate (>99%, extra pure) and ferrous sulfate (99.5%) were purchased form Acros organics (Geel, Belgium). Chloroform (≥99%) was purchased from Carl Roth GmbH + Co. KG (Karlsruhe, Germany) and anhydrous sodium carbonate from Scharlab S.L. (Sentmenat, Spain).

### 2.2 Fish sample preparation

Fresh herring (*Culpa harengus*) frame and salmon (*Salmo salar*) head were provided by Sweden Pelagic AB (Ellös, Sweden) and Fisk Idag AB (Gothenburg, Sweden), respectively. The raw materials were covered with ice-filled plastic bags and arrived tree hours later in the marine lab at Chalmers University of Technology. Using a tabletop mincer equipped with a hole plate having 4.5 mm holes (C/E22 N, Minerva, OMEGA group s.r.L., 40138 Bologna, Italy) the raw materials were grinded and then packaged into plastic zip lock bags, where the air was manually squeezed out. The mince was stored at −80 °C until further use.

### 2.3 Protein isolation using classic pH-shift processing without and with ultrasound

The minced raw materials were subjected to alkaline pH-shift processing following the main principle reported by Abdollahi and Undeland [14] with some modifications. First, 100 g or 500 g of minced raw material was homogenized with 3, 4 or 6 parts of cold distilled water for 1 min at 6000 rpm using a Silverson L5M homogenizer (Silverson Machines Ltd, England). The pH of the homogenate (H) was adjusted to pH 11.5 using 2 M sodium hydroxide (NaOH). After 10 min incubation while stirring, the homogenate was centrifuged at 4000 × g in a Thermo Scientific Sorvall LYNX 6000 Centrifuge (Thermo Scientific, Germany) for 10 min at 4 °C. Thereafter, the middle phase - referred to as first supernatant (S1) - containing the solubilized proteins was separated from the floating lipid layer and the insoluble residues such as skin and bones by pouring it through a metal sieve. The pH of the supernatant was adjusted to pH 5.5 using 2 M hydrochloric acid (HCl) and after 10 min incubation, a second centrifugation step followed (4000 × g, 4 °C, 10 min) to separate processing water (S2) from the protein isolate (PI). The process was either stopped here, for yield analysis, or it was continued with collection of the PIs for subsequent quality analysis. For the latter, the protein was dewatered using a third centrifugation step at 8000 × g for 10 min (4 °C) and the moisture content was adjusted to 80% ± 1% by adding distilled water or a further centrifugation. Lastly, the pH of the PI was adjusted to pH 7 using 2 M NaOH. The entire process was conducted on ice. The PI was stored at −80 °C until further use.

**Fig. 1:**
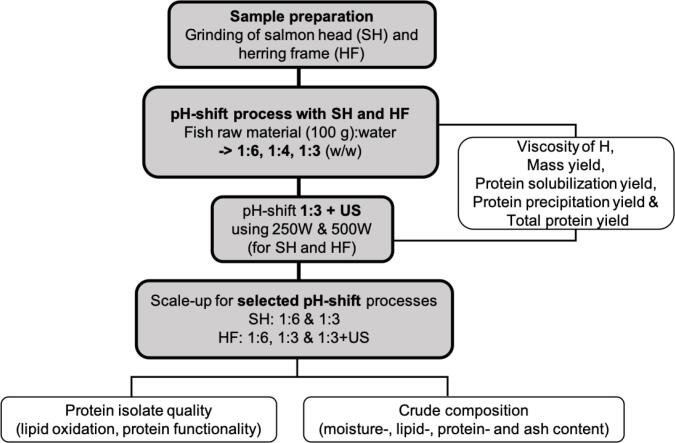
Schematic overview of the workflow. US: additional ultrasound step with a power setting of 250 W or 500 W. H: Homogenate. In the scale-up 500 g raw material were used and US was set at 250 W.

For yield analysis, the pH-shift process was run in duplicates (n = 2) using 100 g of fish raw material. To evaluate the crude composition and PI quality (functional properties and lipid oxidation), one pH-shift process was conducted with 500 g minced raw materials (n = 1). A schematic overview of the workflow of the different pH-shift processes can be seen in Fig. 1.

To compensate for the observed loss in protein solubility and protein yield when reducing the raw material-to-water ratio, an additional step comprising US was introduced for the pH-shift process run with a water ratio of 1:3. After adjusting the pH to 11.5, the homogenate was subjected to US using a probe ultrasonicator (UIP1000hdT, Hielscher Ultrasonics GmbH, Germany), with a titanium probe having a tip diameter of 22 mm and operating at a frequency of 20 kHz. The following settings were chosen when pH-shift processing 100 g of either SH or HF; A US power of 250 W or 500 W was set, while not actively changing the amplitude. In a 600 mL glass beaker, the homogenate was treated for 20 min, using a cycle of pulses of 10 s on and 40 s off, resulting in a total time of US of 4 min. The process was conducted on ice while stirring with a magnet stirrer. After the US treatment, the homogenate was centrifuged and the additional steps were carried out as described above.

US-aided pH-shift processes were run at least in duplicate (n ≥ 2). After the yield calculations were completed, upscaling of the pH-shift process to 500 g raw material was done for HF (n = 1) using the power settings of 250 W. For upscaling, a five L plastic beaker and an overhead stirrer were used instead of a magnet stirrer. The ultrasound intensity (UI) was approximated by the calorimetric method described by Hagenson and Doraiswamy [15].

### 2.4 Analysis of homogenate viscosity

The viscosity of H after the first pH adjustment or after the US step was measured using roughly one mL of sample that was loaded onto the modular compact rheometer (Paar Physica, Rheometer MCR 300, Anton Paar GmbH, Austria). The viscosity over time was recorded using a parallel plate (diameter: 25 mm) on rotation mode with an increasing share rate from 0.01 to 100 1/s log at 10 °C over an interval duration of 600 s. The initial viscosity of H from the pH-shift process performed with 100 g raw material was used for the comparison (n = 2).

### 2.5 Protein and mass yield

To analyze the protein solubilization yield, protein precipitation yield and total protein yield over the pH-shift process (with or without US) samples of H, S1 and S2 were taken and their protein content was measured in triplicates (n = 3) using the Lowry method as modified by Markwell [16], [17]. The equations (1)–(3), listed below were used to calculate the solubilization yield, precipitation yield and total yield of proteins in the process [5], [12].

(1) $$Solubilization\;yield\;\left[\%\right]=\frac{\left(Protein\;content\;of\;S1\;\left[\frac{\mathrm{mg}}{\mathrm{mL}}\right]\;\times \;volume\;S1\;\left[mL\right]\right)}{\left(Protein\;content\;of\;H\;\left[\frac{\mathrm{mg}}{\mathrm{mL}}\right]\;\times \;volume\;H\;\left[mL\right]\right)}\times 100$$

(2) $$Precipitation\;yield\;\left[\%\right]=\frac{\left(Protein\;content\;of\;S1\;\left[\frac{\mathrm{mg}}{\mathrm{mL}}\right]\;\times \;volume\;S1\;\left[mL\right]\right)-\left(Protein\;content\;of\;S2\;\left[\frac{\mathrm{mg}}{\mathrm{mL}}\right]\;\times \;volume\;S2\;\left[mL\right]\right)}{\left(Protein\;content\;of\;S1\;\left[\frac{\mathrm{mg}}{\mathrm{mL}}\right]\;\times \;volume\;S1\;\left[mL\right]\right)}\times 100$$

(3) $$Total\;yield\;\left[\%\right]=\frac{Protein\;content\;S1\;\left[mg\right]-Protein\;content\;of\;S2\;\left[mg\right]}{\mathrm{Protein content of H}\;[mg]}\times 100$$

To calculate the mass yield, the weight of the PI was first standardized to the moisture content of the minced raw material, then the mass yield was determined by dividing the moisture-standardized PI by the initial weight of the raw material used in the process (Equation(4)).

(4) $$\mathrm{Massyield}\;[\%]=\frac{\mathrm{moisture}-standardized\;protein\;isolate\left(PI\right)\;\left[g\right]}{\mathrm{initial raw material}\;[g]}\times 100$$

### 2.6 Crude composition analysis

Total protein content of the raw materials and their corresponding protein isolates was determined at least in duplicates (n ≥ 2) with the Dumas method in an Elementar vario MICRO cube using 2–5 g of freeze-dried sample. A nitrogen-to-protein conversion factor of 5.58 was used [18].

Lipid extraction and gravimetric lipid content measurement was performed in duplicates (n = 2) according to Lee *et al.* [19] as modified by Undeland *et al.* [20]. For both HF and SH, a chloroform:methanol ratio of 2:1 (v/v) was used, while for HF PIs a ratio of 1:2 (v/v) and for SH PIs a ratio of 1:1 (v/v) was used based on their lower lipid contents. Moisture and ash contents were determined gravimetrically in triplicates (n = 3) as described by Abdollahi and Undeland [5] after drying at 105 °C for 24 h and burning at 550 °C for six h, respectively.

### 2.7 Protein quality analysis

#### 2.7.1 Polypeptide pattern analysis using SDS-PAGE

The polypeptide pattern of the mince and PI of SH and HF was investigated using SDS-PAGE according to the method of Laemmli [21] described by Abdollahi and Undeland [14]. The following modifications were made: first, 12 mL of 5% SDS solution were added to 1.3 g of each sample and homogenized using an IKA polytron (T18, digital ULTRA TURRAX®, Germany) at 6000 rpm for one min. The proteins were then dissolved by heating the homogenate in a water bath for one hour at 85 °C. The protein content was measured using the Lowry protein determination modified to Markwell as described above and adjusted to 4 μg protein/mL. Afterward, 50 μL of the sample were mixed with an equal amount of Laemmli buffer (Bio-Rad, USA) containing 5% Beta-mercaptoethanol (2-Mercaptoethanol, Bio-Rad, China), heated at 95 °C for five minutes and then cooled, before centrifuging the sample for five minutes at 5000 × g (Heraeus Fresco 17, Thermo Fisher Scientific, Germany).

For the gel electrophoresis, 20 µg protein of each sample was loaded onto a precast gel (Mini-PROTEAN TGX Gels, Bio-Rad, USA) and run at 125 V using the Mini PROTEAN ® Tetra cell (Bio-Rad, USA) and 1X TGS Buffer. As a standard, five μL of ladder (Precision Plus, Protein TM Dual color, Standards, 10 – 250 kDa, Bio-Rad, USA) was used. The SDS-PAGE gel was stained for 30 min with a staining solution containing 0.02% (w/v) Coomassie® Brilliant Blue G-250 (Bio-Rad, USA) in 50% (v/v) methanol and 7.5% (v/v) acetic acid. The gel was then destained twice with a destaining solution containing 50% (v/v) methanol and 7.5% (v/v) acetic acid, for 30 min. The gel was left in the fridge overnight before scanning and analyzing the bands with a GS-800 Calibrated Densitometer (Bio-Rad, USA).

#### 2.7.2 Rheological characterization of protein isolates

To analyze the dynamic viscoelastic properties of the PIs, they were subjected to an *in situ* gelation, similar as described by van Berlo *et al.* [22]. Frozen PIs were thawed under cold running tap water and directly chopped on ice for 30 s in a small chopper (Black and Decker®, China), followed by adding 2 % (w/w) of NaCl and then chopping the sample for two more minutes to create a homogenous paste. For the analysis, 1–2 g of the paste (n = 1) was loaded on a dynamic rheometer (Paar Physica, Rheometer MCR 300, Anton Paar GmbH, Austria). A parallel-plate (Anton Paar GmbH, Austria) with a 25 mm plate diameter and a 1 mm plate gap as well as an oscillating mode was used to identify the viscoelastic properties. To prevent evaporation, the exposed edge of the sample was covered with mineral oil. The *in situ* gelation was done in three steps in a viscoelastic region (1% strain, 0.1 Hz frequency). First, the temperature increased from 20 °C to 90 °C at a constant heating rate of 5 °C/min, after which the highest temperature was held for 30 min, before ramping the temperature down to 20 °C at a constant cooling rate of 5 °C/min.

#### 2.7.3 Gel preparation from protein isolates

Gel production was done similarly to what was described by van Berlo *et al.* [22]. Briefly, after producing a homogenous PI paste, as described in 2.7.1., the paste was transferred to 20 mL syringes (BD Plastipak, Becton Dickinson S.A., Madrid, Spain) and sealed. Thereupon, the paste was subject to a two-step gelation: first, in a 35 °C water batch for 30 min, followed by 20 min in a 90 °C water bath. Immediately after, the syringes were cooled down using ice and were then stored in the fridge (4 °C) overnight to be analyzed the next day.

#### 2.7.4 Texture profile analysis (TPA) of gels

Texture properties of the gel such as gumminess, springiness, chewiness, cohesiveness and firmness were analyzed as explained by Abdollahi *et al.* [23]. The gel was cut into five cylindrically shaped pieces with a diameter and height of ∼ 1.7 cm. Following equilibration to room temperature (RT), the gel samples were subject to texture profile analysis (TPA) using a texture analyzer TVT 6700 (Perten Instruments, PerkinElmer company, Australia). With help from a compression plate (Perten Instruments, N672040, TVT, diameter 44 mm, stainless steel), the gel was subjected to compression (40%) twice with five s rest in-between the compression cycles, at a depression speed of 60 mm/min. Five replicates were made for each gel (n = 5).

#### 2.7.5 Color measurement of gels

The surface color of the freshly cut protein gels (diameter ∼ 1.7 cm) was measured with a colorimeter (Croma Meter CR-400, Konica Minolta, Japan) as explained by Abdollahi and Undeland [14].

#### 2.7.6 Water holding capacity (WHC) of gels

The water holding capacity (WHC) of gels was measured according Abdollahi *et al.* [23]. Two g of gel sample (*Ws*) were chopped and wrapped into pre-weighed (*Wi*) filter paper (Munktell filter paper No3, Munktell filter AB, Grycksbo, Sweden) resulting in at least five layers of filter paper. The sample was placed inside a 50 mL Falcon tube and centrifuged in a tabletop centrifuge (Multifuge 1 s, Heraeus, Germany) at 3000 × g for 10 min at RT. Afterward, the sample was removed and the filter paper was weighed again (*Wf*). The experiment was run in triplicates (n = 3). Equation (5) was used to calculate WHC [%] in which *M* is the initial moisture content [%] in the sample. Note that for SH gel samples, the experiment was not suited to measure WHC accurately, as they were not solid enough.

(5) $$\mathrm{WHC}\left[\%\right]=\frac{\mathrm{Ws}\times \left(\frac{M}{100}\right)-\left(Wf-Wi\right)}{\mathrm{Ws}-\left(\frac{M}{100}\right)}\times 100$$

#### 2.7.7 Lipid oxidation analysis

The degree of lipid oxidation of the raw materials and their PIs was analyzed by measuring their level of free thiobarbituric acid reactive substances (TBARS) using the method described by Schmedes and Hølmer [24]. The TBARS were analyzed in the methanol/water phase that was set aside from the lipid extraction. From each of the two lipid extractions done from each sample type, duplicate analyses were made. For quantification, a standard curve was made from malondialdehyde (MDA).

### 2.8 Statistical analysis

Statistical analysis was performed using the SPSS software (IBM Corp. Released 2021. IBM SPSS Statistics for Macintosh, Version 28.0. Armonk, NY: IBM Corp). The data were evaluated using a one-way analysis of variance (ANOVA) followed by Duncan’s multiple range test to determine significant differences between treatment groups. All statistical analyses were performed with a significance level of 0.05, where differences of p < 0.05 were considered significant. The results were reported as mean ± standard deviation (SD) (n ≥ 2).

## 3 Results and discussion

### 3.1 Viscosity of homogenate at solubilization pH

To assess the effect of the different water ratios as well as the additional US step on the pH-shift process, the viscosity of the homogenate was measured since it can affect separation efficiency during the first centrifugation step. Reducing the water ratio increased the viscosity of both the SH and HF homogenate at solubilization pH (Figure A 1, supplementary data). This increase reflects the lower dilution of proteins and other constituents.

An additional US step (250 W and 500 W) slightly increased the viscosity of SH homogenized with 3 ratios of water (p > 0.05). An opposite effect was observed in HF, where the viscosity significantly (p < 0.05) decreased compared to the pH-shift process with the same water ratio (1:3) without US. This contradictory effect of US on viscosity of the homogenate in the two studied biomasses could be related to induction of protein aggregation and protein solubilization in SH and HF homogenates, respectively. One reason for protein aggregation induced by US could be possible radical formation (e.g. H or OH radicals) during sonication [25]. These radicals can undergo additional reactions resulting in the generation of reactive oxygen species (ROS). ROS may then react with the proteins, producing protein radicals which can generate protein crosslinking [26] and subsequent protein destabilization, ultimately leading to aggregation. For instance, Gülseren *et al.* [27] showed that in a bovine serum albumin solution, high-intensity US increased particle size and decreased free sulfhydryl groups, which might be attributed to protein aggregation. Another reason for increased viscosity could be the re-aggregation of disordered proteins via hydrophobic bonds. Hydrophobic interaction as a driving force for aggregation was also suggested by Arzeni *et al.* [28], who reported increased particle sizes of egg white proteins after US treatment. Conversely, for HF the cavitation phenomenon could have promoted proteins to become more charged, making protein–protein repulsion predominant and increasing protein-water interactions [2]. The repulsion forces caused by the highly charged proteins could then expand further until they could not be sustained. This would dissociate the proteins, leading to smaller fragments and subsequently a decreased viscosity. Moreover, the US might have led to increased shear forces between solvent and polymers that result in the breaking of protein bonds, increasing the protein solubility. In this way, large proteins like titin and nebulin might be broken down and contribute to the decrease in viscosity. A similar mechanism was proposed by Tian *et al.* [13], where the addition of US also led to a decrease in consistency for alkaline pH-shift processed tilapia fillets. In several studies [12], [29], [30], [31] US treatment has been shown to decrease particle size, which subsequently could decrease viscosity and enhance solubility.

The different effects of US on the two different raw materials might be attributed to their different compositions in terms of lipid, bone and soluble protein content. Different muscle proteins i.e., sarcoplasmic and myofibrillar proteins, may also react differently to the cavitation energy. The former can be confirmed in the polypeptide pattern, which visibly shows higher intensity of myosin heavy chain (MHC) bands for HF than for SH, indicating that there are more myofibrillar proteins present in HF than in SH (Fig. 3). Gaining more insight into what effect US has on different muscle proteins might facilitate optimizing the US treatment for different types of raw materials. Generally, the decrease in viscosity indicated that US might have increased the solubility of the proteins, which was later verified (see section 3.2).

**Fig. 2:**
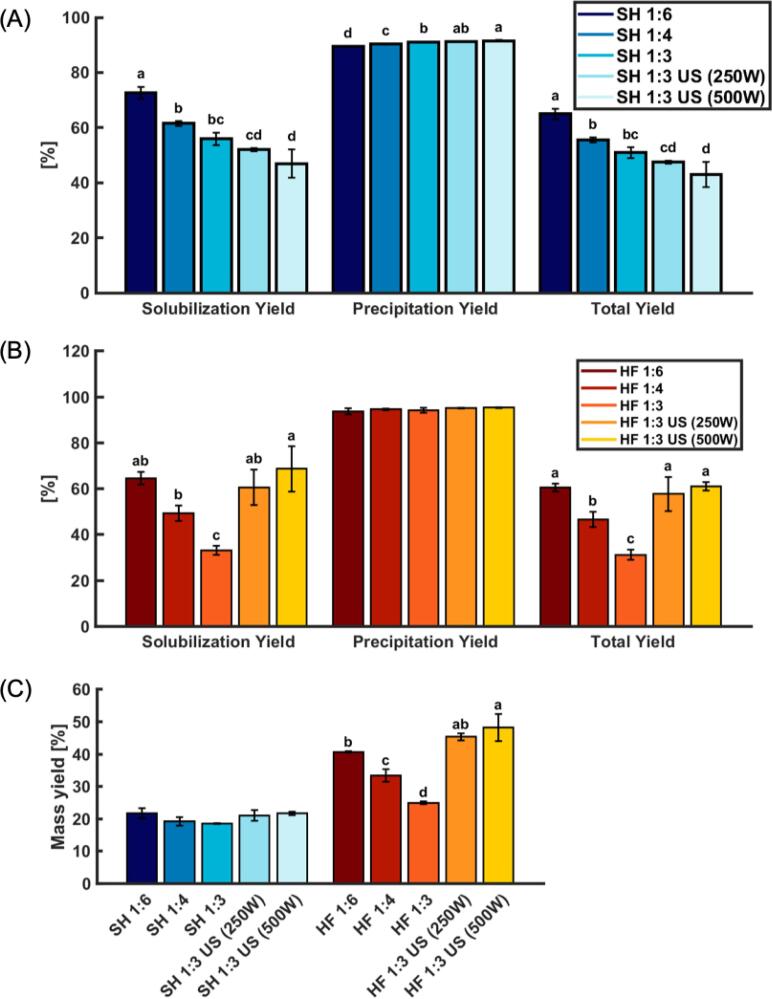
Effect of different water ratios and additional ultrasound (US) on solubilization/precipitation/total protein yields during pH-shift processing of salmon head (SH) (A) and herring frame (HF) (B). Panel (C) shows mass yield [%] for HF and SH as a function of the different processes. 1:6, 1:4 and 1:3: Raw material-to-water ratios used in the pH-shift process. US(W): additional ultrasound step with a power setting of 250 W or 500 W. The data are shown as mean values (n ≥ 3) with the error bar indicating ± SD. The different small letters indicate a significant difference (p < 0.05) within one type of raw material.

**Fig. 3:**
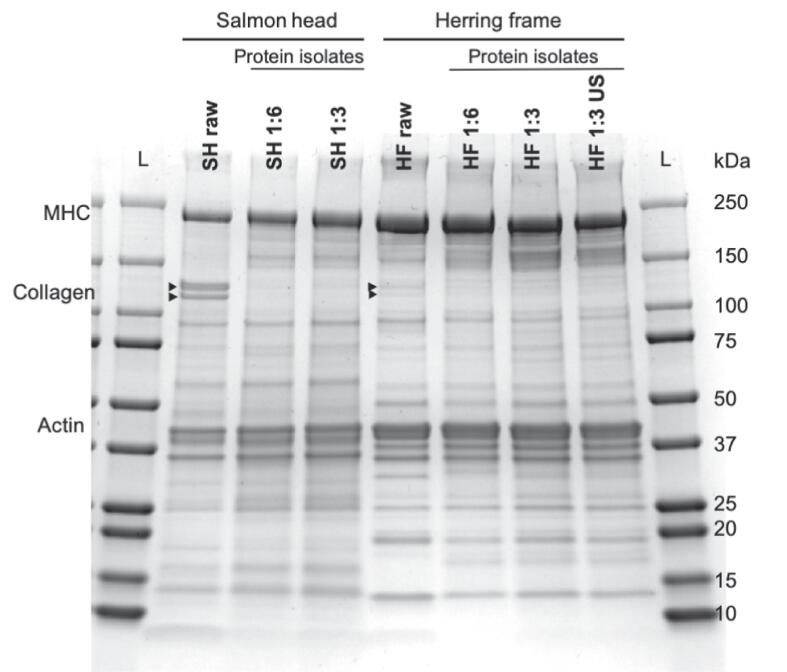
Polypeptide pattern of salmon head (SH) and herring frame (HF) and their protein isolates. Raw: Initial minced raw material, 1:6 and 1:3: Raw material-to-water ratio used in the pH-shift process. US (250 W): Additional ultrasound step with a power setting of 250 W. MHC: myosin heavy chain. As ladder (L) the Precision Plus (Protein TM Dual color, Standards, Bio Rad) was used. 20 µL of protein were loaded on to the gel.

### 3.2 Protein solubility and yield

Protein solubility is regarded as one of the major factors affecting protein yield during pH-shift processing [7]. For this reason, the effects of water ratio and US on protein solubilization yield, protein precipitation yield and total protein as well as mass yield were assessed (Fig. 2).

Decreasing the water ratio decreased protein solubilization yield and total protein yield for both SH and HF. A similar trend in solubility was also reported by Chomnawang and Yongsawatdigul [8] when using water ratios 1:9, 1:7, 1:5 and 1:3 for tilapia frame side streams. For SH there was a significantly (p < 0.05) higher protein precipitation yield at decreased water ratios. However, as it did not result in a higher total protein yield, it was not further investigated. In HF there were no significant (p > 0.05) differences between the precipitation yields as water ratios were varied.

Similar to the effects on viscosity, US had a contrary effect on SH compared to HF. When adding US, regardless of the power setting, the solubilization yield and total yield decreased in SH, whereas in HF, these parameters significantly (p < 0.05) increased. As previously stated, US can lead to higher shear forces, which can decrease particle sizes, resulting in a larger contact area between the protein particle and water [13], [32]. Furthermore, the cavitation phenomenon creates local “hot spots” with high temperatures and pressure that can lead to unfolding and hydrolysis of proteins. These changes in conformation and polypeptide size can expose previously hidden hydrophilic groups which further enhance protein-water interaction and hence increase solubility [33]. Also, an increase in temperature might facilitate the solubilization of the proteins [34]. An increase in solubilization- and precipitation yield when adding US was in line with previous findings from Pezeshk *et al.* [12] when pH-shift processing side streams from rainbow trout (head + tail-on backbone) and from Tian *et al.* [13] when processing tilapia fillets. It is noteworthy that their findings were achieved when using a water ratio of 1:6 and 1:9, respectively. Hence, the present study confirms that depending on side stream US can increase yield even at a low water ratio of 1:3 allowing to use lower water ratio in the pH-shift process without jeopardizing the yield. For HF the increase in solubilization yield and total protein yield due to US are also believed to explain the increase in mass yield (Fig. 2C).

That US was not able to compensate for the negative effect of the water ratio on SH protein solubilization yield and total protein yield could be due to increased exposure of hydrophobic groups which re-aggregates the proteins and decreases solubility [28]. Such a decrease in solubility at relatively high intensity of US treatment (107 W/cm^2^) was reported by Shen *et al.* [35] for whey protein isolates. Similarly, Arzeni *et al.* [28] reported a decrease in solubility of egg white proteins when treating them with US, attributed to aggregation. Aggregates formed in the solubilization step will then be precipitated in the first centrifugation, reducing protein recovery in the actual precipitation step, and hence reducing total protein yield. Likely the decrease in protein solubility due to formation of small aggregates led to the observed increase in viscosity.

As previously hypothesized, the contrasting effects of US on the two raw materials might be due to their different ratios between myofibrillar and sarcoplasmic proteins and it could be that these groups behave differently when subjected to US. Based on the polypeptide pattern, HF has more myofibrillar proteins than SH (Fig. 3). That the raw materials used by Pezeshk *et al.* [12] and Tian *et al.* [13], rainbow trout head + tail-on backbone and boneless tilapia fillets, also most likely contained a large ratio of myofibrillar proteins supports the hypothesis that myofibrillar proteins behave more favorable to US compared to sarcoplasmic proteins. However, more research would be needed to verify this hypothesis and also whether the US setting used was more suitable for HF than SH. By correctly tuning the US treatment, the beneficial effects on protein yields might also be seen in SH.

It was striking that there were no significant (p > 0.05) differences in viscosity or yield between the two different US power settings (250 W and 500 W). However, when calorimetrically measuring the power that is dissipated from the sonotrode (titanium probe), it was discovered that both power settings resulted in almost the same amount of power being dissipated into the system and consequently yielding the same UI. While it is known that the effect of ultrasound on protein yield at different powers does not follow linearity and several factors (e.g. amplitude or temperature) influence power output [36] it is currently not fully understood why the two different settings lead to the same power input to the system.

While there were no significant (p > 0.05) differences in mass yield for SH between the different processes, reducing the water ratio resulted in a significantly (p < 0.05) decreased mass yield for HF (Fig. 2C). This loss in mass yield could, however, be fully compensated when adding an US step at 250 W or 500 W.

Based on these findings, only HF, and only 250 W were used in further investigation of the effect from US on protein isolate quality (functional properties and lipid oxidation) and crude composition.

### 3.3 Crude composition

The crude composition of the minced raw materials and the PIs is shown in Table A 1 (supplementary data). There was no significant (p > 0.05) difference within SH PIs regarding lipid- and ash content. For PIs from HF, there was no significant (p > 0.05) difference in lipid content. However, there was a significant (p < 0.05) difference in ash content between the PI made with 6 compared to those made with 3 parts of water; both with and without US. This is most likely due to the more difficult manual separation of the fractions after the first centrifugation when smaller water volumes were used and/or when the homogenate had a higher viscosity.

The pH-shift process up-concentrated myofibrillar protein from both SH and HF and there were no significant differences (p < 0.05) in PI protein content. This is mostly due to the presence of collagenous proteins in the start materials which has be measured via Dumas method. Overall, the reduction of water and the combination with US did not have any significant (p > 0.05) impact on crude composition of the PI except a slight increase (p < 0.05) in the ash content of the PIs.

### 3.4 Polypeptide pattern

The polypeptide pattern of the minced raw materials and their respective PIs are shown in Fig. 3. Almost all bands seen in the raw materials mince can also be found in the PIs, except for two bands between 100 and 150 kDa that show the collagen subunits α1 and α2. This further underlines the efficacy of the pH-shift process to fractionate the soluble proteins from insoluble materials. In the SH raw material, the collagen bands were more pronounced compared to in HF as the type of raw material is more collagenous. The most abundant polypeptide band in both raw materials and their PIs was MHC (∼220 kDa) followed by actin (∼43 kDa); HF-derived samples however had more intense MHC bands than SH. This could reflect the higher ratio of muscle proteins coming from frame compared to head.

Below the MHC band from the PIs, there was a dark shadow which appears to increase when decreasing the water ratio from 6 to 3 parts, particularly for HF. Most likely this reflects small amounts of enzymatic proteolysis during the pH-shift process [37]. It is well known that pelagic species such as herring have a high enzymatic activity [23]. Previous studies have also found similar polypeptide patterns including a partial degradation of myosin during pH-shift processing; the latter ascribed to proteases [5], [38]. There was no major difference observed in MHC degradation with and without US. Also Pezeshk *et al.* [12] did not see any changes in the polypeptide pattern of rainbow trout when adding US to the pH-shift process.

### 3.5 Rheological characterization

**Fig. 4:**
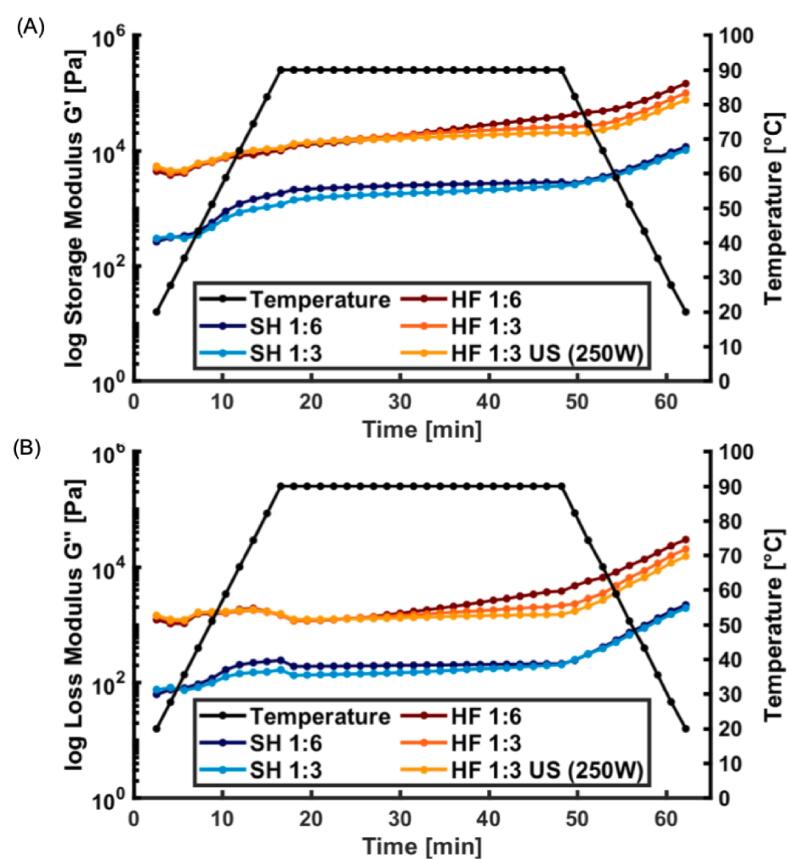
Storage Modulus G' (A) and Loss Modulus G'' (B) over time of protein gels made from salmon head (SH) and herring frame (HF). 1:6, 1:3: Raw material-to-water ratio used in the pH-shift process. US (250 W): Additional ultrasound step with a power setting of 250 W. Single measurement.

The visco-elastic properties of the PIs from SH and HF produced using different process parameters are shown in Fig. 4A and B. Over time both storage modulus (G’) and loss modulus (G’’) increased in all samples. At the beginning of the gelation process, a slight reduction in G’ could however be observed, especially for HF. This softening has earlier been seen for different fish proteins and is related to the denaturation of the myofibrillar proteins and oxidation of sulfhydryl groups [39], [40]. The subsequent increase in G’ reflects the three-dimensional (3D) gel network formation from partially denatured proteins which involves interactions e.g., covalent bonds between myosin tails and hydrophobic interactions between the myosin head portions [41]. During the pH-shift process, the proteins are partially denatured which can result in partly exposed sulfhydryl groups which can facilitate the formation of disulfide bonds that stabilize the gel [23]. Generally, for both SH and HF, G’’ was much lower than G’ indicating a more elastic and less viscous nature of the samples during the *in-situ* gelation. Moreover, gels from SH had a lower G’ and G’’ than HF suggesting a less elastic nature than HF. The higher structure formation and increase in G’ and G’’ from frame compared to head is in line with the higher content of protein, and particularly of MHC, in the former as confirmed by the polypeptide pattern (Fig. 3). During the isothermal step at 90 °C, the network development continued but at slower kinetics. In the cooling, a further increase in G’ and G’’ can then be observed. The latter can be mainly attributed to hydrogen bonds and hydrophobic interactions forming as the gel stabilizes and immobilizes water in the protein network. The final G’ and G’’ of gels from SH remained lower than for HF, showing that head PI had a lower gel-forming capacity than that from the frame raw material. Apart from less MHC in SH, another reason could be that head contain more proteases and other enzymes that might result in reduced gelling properties [42]. Overall, reduction of the water ratio from 6 to 3 and the combination with US did not affect viscoelastic properties of PI from HF during the *in situ* heat induced gelation.

### 3.6 Textural properties of gels

**Fig. 5:**
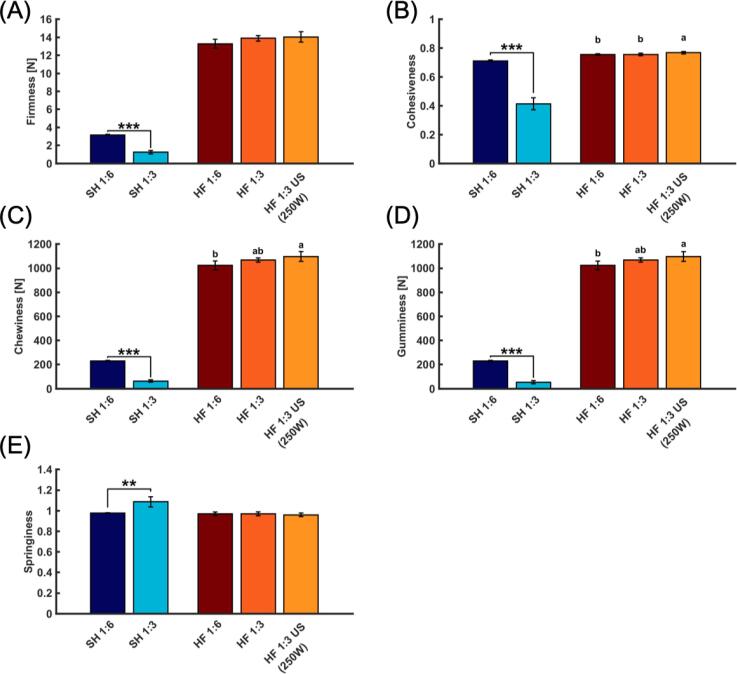
Firmness [N] (A), Cohesiveness (B), Chewiness [N] (C), Gumminess [N] (D) and Springiness (E) of gels made from salmon head (SH) and herring frame (HF) protein isolates (PI). 1:6, 1:3: Raw material-to-water ratio used in the pH-shift process. US (250 W): Additional ultrasound step with a power setting of 250 W. The results are shown as mean (n = 5) with the error bars indicating ± SD. Asterix and the different small letters indicate significant differences (p < 0.05) within one type of raw material.

In the texture profile analysis (TPA), firmness, cohesiveness, chewiness, gumminess and springiness of the PI gels were determined (Fig. 5A-E). When comparing the PIs coming from different raw materials, HF-derived PI reached higher values in four out of five analyses, overall showing that HF PIs resulted in a more elastic gel compared to SH PIs. This is consistent with the results from the rheological analysis. Again, the higher amount of myofibrillar proteins in HF, which are the main drivers for gel formation, is a likely reason [5]. Contrary, lipids and sarcoplasmic proteins, both being enriched in SH PI (Table A 1, Fig. 3), can contribute to weakening of the gel stability. The latter lack gel forming capacity and both may interfere with the actomyosin crosslinking [43]. In line with these findings Abdollahi and Undeland [5] reported higher breaking force in PIs coming from herring than salmon raw materials (head- and tail-on backbone).

Reducing the water ratio from 1:6 to 1:3 significantly (p < 0.05) reduced firmness, gumminess, cohesiveness and chewiness in SH gels. Only the springiness of SH was increased when 3 parts of water were used. Although there were no significant (p > 0.05) differences in lipid, protein or ash content between the two SH PIs, it is still possible that the trend of an increased lipid and ash content in SH PI made with 3 water parts could have influenced the gelling properties. Opposite to SH, reducing the water ratio from 6 to 3 parts when producing PIs from HF did not result in any significant (p > 0.05) difference in the measured textural properties. Also, the additional US step did not significantly (p > 0.05) alter HF PI gel properties, with the exception of cohesiveness. However, since the differences were minor it was not further investigated.

### 3.7 Color

The highest whiteness was measured in protein gels from SH PI with a value of 62 reached when using 6 parts of water and 61.30 with 3 parts. HF gels generally had lower whiteness; 52.60 (1:6), 53.05 (1:3) and 53.17 (1:3 with US) (Figure A 2, supplementary data); the two latter not being significantly different. The lower whiteness in herring PIs is most likely related to a higher amount of heme pigments [44]. It has been shown that the pH-shift process can lead to oxidation of heme pigments converting them to the brown methemoglobin or metmyoglobin [45]. The lower lipid level in HF PI than SH PI may also contribute to its lower whiteness [5].

### 3.8 WHC

The WHC of HF-derived protein gels was not significantly (p > 0.05) affected by decreasing the water ratio nor by the use of an additional US step (Figure A 3, supplementary data). This correlates with the lack of effect from these process parameters on firmness of the gels (Fig. 5A). Compared to reported findings from Abdollahi and Undeland [5] and van Berlo *et al.* [22], WHC of herring PI gels in this study was higher; ∼85–87% vs. 67%. This could be related to the higher content of total protein and MHC, which might have resulted in a better self-supporting 3D gel structure translating into higher WHC. However, the gel structure and its WHC is indeed influenced also by many other factors, for instance the type of protein–protein interactions and protein conformational changes taking place in the gelation process [46]. In line with the lack of effects from US on textural properties, there was no significant (p > 0.05) difference in WHC between HF-derived gels made without and with US.

### 3.9 Lipid oxidation

**Fig. 6:**
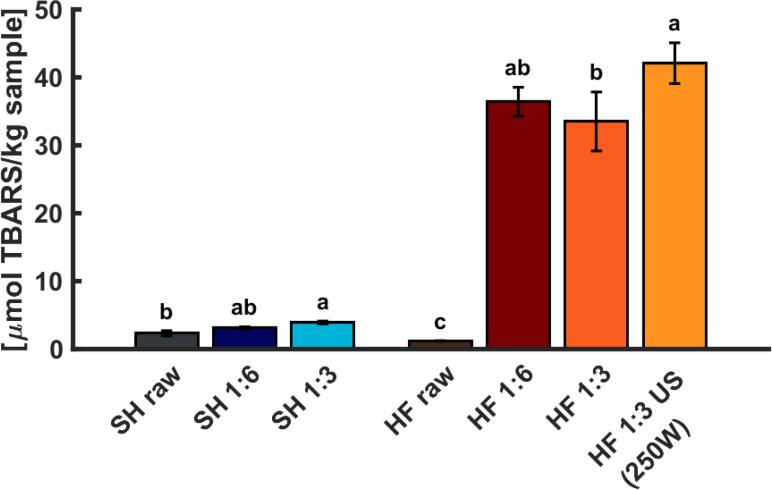
TBARS as indicator for secondary lipid oxidation products in salmon head (SH) and herring frame (HF) as well as their protein isolates. Raw: Initial minced raw material. 1:6 and 1:3: Raw material-to-water ratio used in the pH-shift process. US (250 W): Additional ultrasound step with a power setting of 250 W. Results are shown as mean with error bar indicating ± SD. Different small letters indicate significant differences (p < 0.05) within one type of raw material.

The SH and HF mince and their PIs were assessed regarding their secondary lipid oxidation products measured as TBARS (Fig. 6). The pH-shift process increased TBARS in HF PI compared to the untreated mince, while for SH, the difference was very small, and only significant (p < 0.05) when using 3 parts of water. That herring exhibited greater oxidation compared to salmon was most likely because of the higher hemoglobin (Hb) levels. Hb́s can act as pro-oxidants e.g., by cleaving pre-formed hydroperoxides [47] which consequently can increase TBARS. A similar ranking was reported by Wu *et al.* [44] where herring side streams also reached higher TBARS values than salmon side streams during pH-shift processing.

The reduction of the water ratio did not lead to a significant (p > 0.05) difference in TBARS for either salmon or herring PIs. However, adding an US step during HF processing resulted in significantly (p < 0.05) higher TBARS compared to the same process without US. The decomposition of hydroperoxide to secondary oxidation products is influenced by various factors, for example, temperature, lipid class composition and as mentioned, the presence of pro-oxidants e.g., Hb [48]. Due to the cavitation phenomenon during US treatment, “hot spots” with high temperature and pressure can occur, which might have contributed to an increased breakdown of hydroperoxides. It has also been reported that these extreme conditions can lead to free radicals e.g., hydroxyl radicals [25], [49], which can accelerate lipid oxidation. Chang and Wong [50], found an accelerated biochemical reaction rate as evidenced by TBARS content, when using US to tenderize cobia sashimi. Moreover, Kang *et al.* [51] also discovered an increase in TBARS due to US treatment in beef myofibrillar proteins. Possibly, this process-aid should therefore be combined with an antioxidant addition.

## 4 Conclusions

This study showed that reducing the water ratio from the commonly used 1:6 to a water ratio of 1:3, drastically reduced the protein solubility- and yield of both SH and HF. Altering the water ratio also increased the ash content in PI from HF and led to a significant (p < 0.05) decrease in firmness and cohesiveness of the gels from SH PI. The lower water ratio also resulted in an increased proteolysis of MHC in HF PI. The pH-shift process itself, but not the changed water ratios, increased TBARS values, with a greater impact on herring side streams.

US-aided pH-shift processing successfully enabled to compensate for the loss of yield coming from reducing the water ratio for HF, but not for SH. Most likely this is caused by the difference in composition between the two raw materials and particularly their myofibrillar- and sarcoplasmic protein concentrations. US did not impair the functional properties of HF protein. The only side effect of using US in combination with the lower water ratio was an increase in lipid oxidation ascribed to radical formation or the cavitation phenomenon.

Altogether, using US during pH-shift processing enables half the amount of water to be used while achieving the same protein yield and quality as in the conventional pH-shift process. However, the US condition needs to be carefully tuned for each type of raw material to ensure a positive effect.

### CRediT authorship contribution statement

**Michaela V. Santschi:** Data curation, Investigation, Methodology, Visualization, Writing – original draft. **Ingrid Undeland:** Writing – review & editing, Supervision, Funding acquisition, Project administration. **Mehdi Abdollahi:** Conceptualization, Supervision, Writing – review & editing, Funding acquisition, Project administration.

## Declaration of Competing Interest

The authors declare the following financial interests/personal relationships which may be considered as potential competing interests: Mehdi Abdollahi reports financial support was provided by Sweden’s Innovation Agency. Ingrid Undeland reports financial support was provided by Horizon Europe.
