# Supplementary material for: Ultrasound-aided pH-shift processing for resource-smart valorization of salmon and herring side streams
Source: Ultrason Sonochem. 2023 Aug 1;99:106539. doi: 10.1016/j.ultsonch.2023.106539 (PMC10432243; doi:10.1016/j.ultsonch.2023.106539)
Supplement: Supplementary Data 1 [file mmc1.docx]

# Appendix

Supplementary data


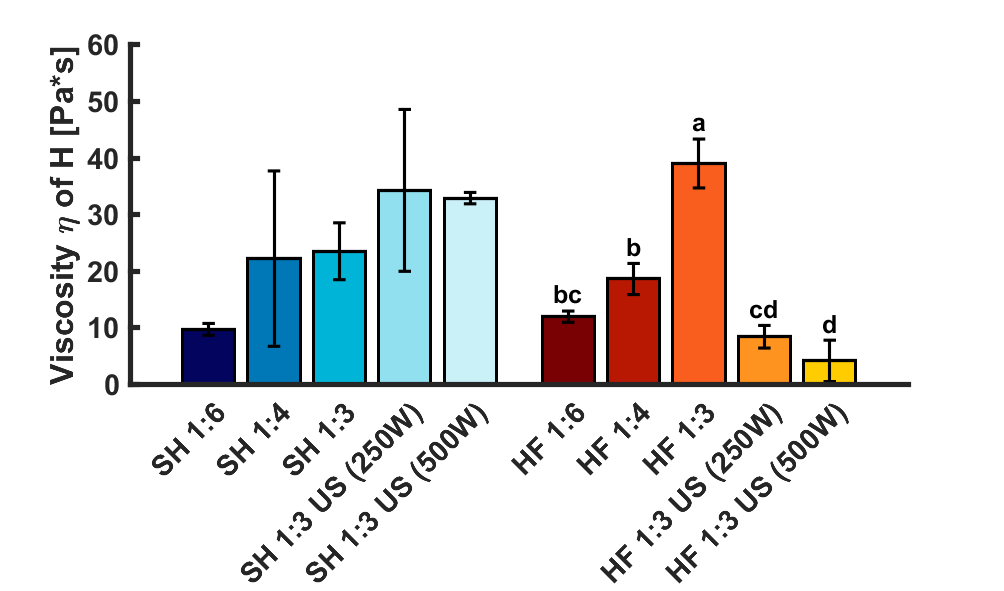


**Figure A 1:** **Viscosity of homogenate (H) of salmon head (SH) and herring frame (HF) at pH 11.5 using classic pH-shift process with different water ratios (1:6, 1:4, 1:3) and with an additional ultrasound (US) at 250W or 500W.** Data are shown as mean (n≥2) with the error bar indicating ± SD. Different small letters indicate a significant difference (p<0.05) within one type of raw material.

**Table A 1:** **Crude composition [%] of salmon head and herring frame and their respective protein isolates (PI) from different pH-shift processes**. 1:6, 1:4 and 1:3: Raw material-to-water ratio used in the pH-shift process. US (W): Additional ultrasound step with a power setting of 250W. Moisture- and lipid content are presented as % wet weight [ww], protein- and ash content are presented based on % dry weight [dw]. The results are shown as mean (n≥2) ± SD. Different small letters in a column show significant differences (p<0.05) within one type of raw material.

| **Species** | **Sample** | **Moisture [%ww]** | **Lipid  [%ww]** | **Protein**  **[%dw]** | **Ash  [%dw]** |
| --- | --- | --- | --- | --- | --- |
| Salmon | Head (minced) | 61.21 ± 0.74^c^ | 21.43 ± 1.60^a^ | 36.81 ± 7.29 | 8.12 ± 0.45^a^ |
|  | PI from 1:6 | 79.05 ± 0.52^b^ | 10.83 ± 0.82^b^ | 41.74 ± 1.18 | 1.74 ± 0.06^b^ |
|  | PI from 1:3 | 80.47 ± 0.12^a^ | 11.10 ± 0.76^b^ | 42.97 ± 1.89 | 2.09 ± 0.11^b^ |
| Herring | Frame (minced) | 77.34 ± 0.38^c^ | 5.16 ± 0.30^a^ | 63.31 ± 8.88 | 12.08 ± 0.07^a^ |
|  | PI from 1:6 | 81.46 ± 0.31^a^ | 2.25 ± 0.29^b^ | 77.20 ± 0.75 | 2.62 ± 0.22^c^ |
|  | PI from 1:3 | 80.04 ± 0.37^b^ | 2.27 ± 0.12^b^ | 77.14 ± 0.12 | 3.69 ± 0.23^b^ |
|  | PI from 1:3 US (250W) | 80.24 ± 0.20^b^ | 2.86 ± 0.17^b^ | 74.94 ± 0.08 | 3.81 ± 0.37^b^ |


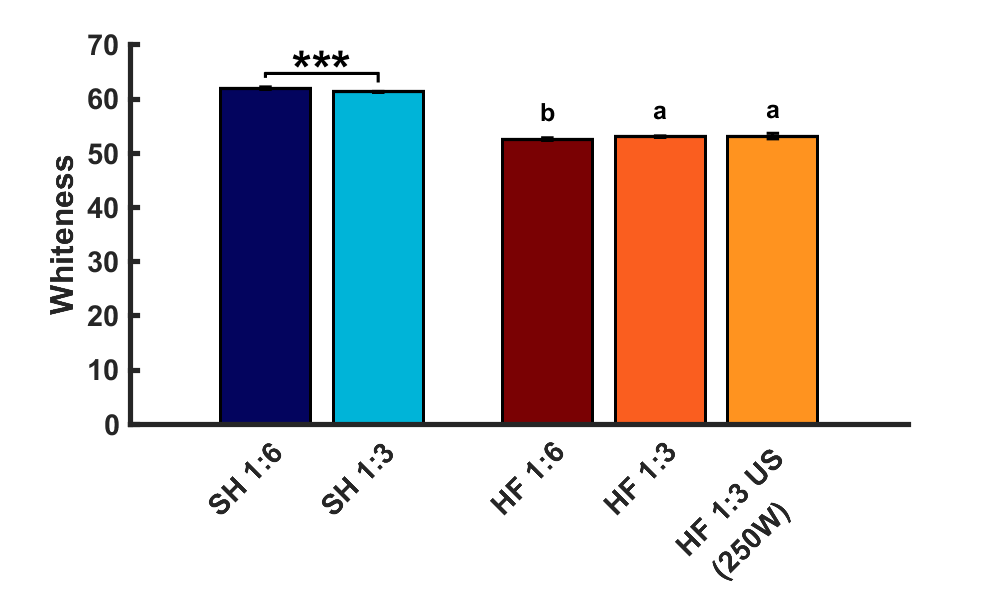


**Figure A 2:** **Whiteness of gels from salmon head (SH) and herring frame (HF) protein isolates (PI).** 1:6, 1:3: Raw material-to-water ratio used in the pH-shift process. US (250W): Additional ultrasound step with a power setting of 250W. Results are shown as mean (n≥7) with the error bar indicating ± SD. Asterix and different small letters indicate significant differences (p<0.05) within one type of raw material.


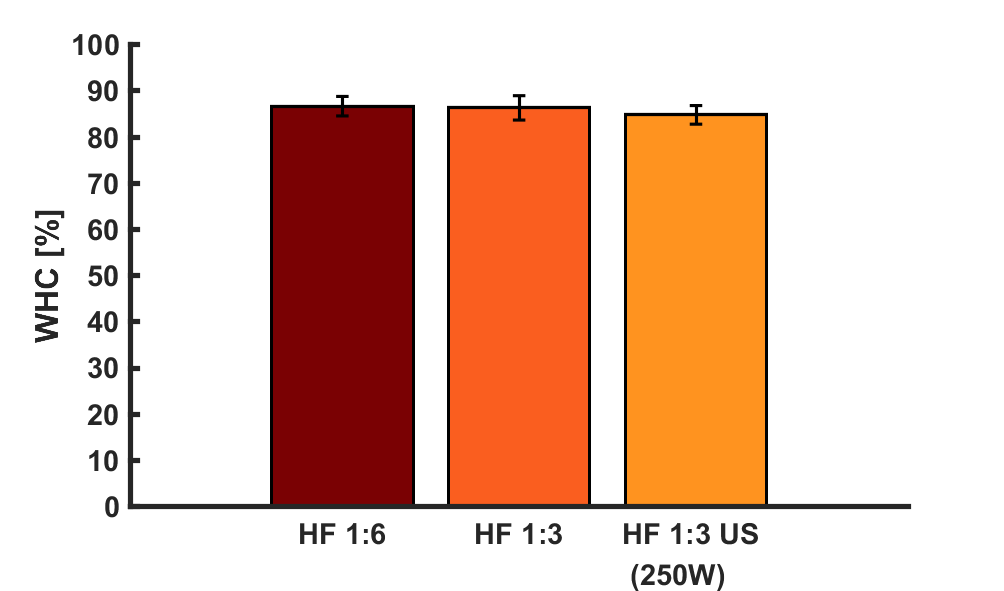


**Figure A 3:** **Water holding capacity (WHC) of gels from herring frame (HF) protein isolate (PI).** 1:6, 1:3: Raw material-to-water ratio used in the pH-shift process. US (250W): Additional ultrasound step with a power setting of 250W. Results are shown as mean (n=3) with the error bar indicating ± SD. There were no significant differences (p>0.05) between the protein gels.
